# 1,3-Bis(prop-2-yn­yl)-1*H*-1,3-benzimid­azol-2(3*H*)-one

**DOI:** 10.1107/S1600536811012578

**Published:** 2011-04-13

**Authors:** Younes Ouzidan, Youssef Kandri Rodi, Jerry P. Jasinski, Raymond J. Butcher, James A. Golen, Lahcen El Ammari

**Affiliations:** a Laboratoire de Chimie Organique Appliquée, Université Sidi Mohamed Ben Abdallah, Faculté des Sciences et Techniques, Route d’Immouzzer, BP 2202 Fès, Morocco; b Department of Chemistry, Keene State College, 229 Main Street, Keene, NH 03435-2001, USA; c Department of Chemistry, Howard University, 525 College Street NW, Washington, DC 20059, USA; d Laboratoire de Chimie du Solide Appliquée, Faculté des Sciences, Université Mohammed V-Agdal, Avenue Ibn Battouta, BP 1014, Rabat, Morocco

## Abstract

In the title compound, C_13_H_10_N_2_O, the fused-ring system is essentially planar, the largest deviation from the mean plane being 0.015 (1) Å. The two propynyl groups are nearly perpendicular to the benzimidazole plane, making dihedral angles of 85 (3) and 80 (2) °, and point in opposite directions. There are two short inter­molecular C—H⋯O contacts to the carbonyl O atom, one involving the acetyl­enic H atom and the other a H atom of the methyl­ene group.

## Related literature

For applications of benzimidazole compounds, see: Gravatt *et al.* (1994 ▶); Horton *et al.* (2003 ▶); Kim *et al.* (1996 ▶); Roth *et al.* (1997 ▶); Ouzidan *et al.* (2011**a* ▶,b* ▶).
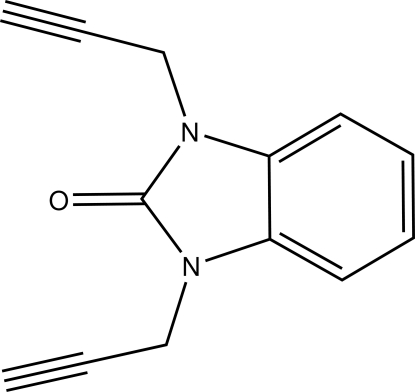


## Experimental

### 

#### Crystal data

C_13_H_10_N_2_O
*M* *_r_* = 210.23
Monoclinic, 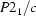

*a* = 7.7398 (4) Å
*b* = 17.1869 (9) Å
*c* = 8.4856 (5) Å
β = 101.459 (6)°
*V* = 1106.28 (10) Å^3^
*Z* = 4
Mo *K*α radiation
μ = 0.08 mm^−1^
*T* = 170 K
0.42 × 0.41 × 0.20 mm

#### Data collection

Oxford Diffraction Xcalibur E Gemini diffractometer
Absorption correction: multi-scan (*CrysAlis PRO*; Oxford Diffraction, 2009 ▶) *T*_min_ = 0.966, *T*_max_ = 0.984
5295 measured reflections
2631 independent reflections
2244 reflections with *I* > 2σ(*I*)
*R*_int_ = 0.014

#### Refinement

*R*[*F* ^2^ > 2σ(*F* ^2^)] = 0.040
*wR*(*F* ^2^) = 0.104
*S* = 1.05
2631 reflections
146 parameters
H-atom parameters constrained
Δρ_max_ = 0.19 e Å^−3^
Δρ_min_ = −0.16 e Å^−3^

### 

Data collection: *CrysAlis PRO* (Oxford Diffraction, 2009 ▶); cell refinement: *CrysAlis PRO*; data reduction: *CrysAlis PRO*; program(s) used to solve structure: *SHELXS97* (Sheldrick, 2008 ▶); program(s) used to refine structure: *SHELXL97* (Sheldrick, 2008 ▶); molecular graphics: *SHELXTL* (Sheldrick, 2008 ▶); software used to prepare material for publication: *SHELXTL*.

## Supplementary Material

Crystal structure: contains datablocks I, global. DOI: 10.1107/S1600536811012578/gk2361sup1.cif

Structure factors: contains datablocks I. DOI: 10.1107/S1600536811012578/gk2361Isup2.hkl

Additional supplementary materials:  crystallographic information; 3D view; checkCIF report

## Figures and Tables

**Table 1:** Hydrogen-bond geometry (Å, °)

*D*—H⋯*A*	*D*—H	H⋯*A*	*D*⋯*A*	*D*—H⋯*A*
C8—H8*A*⋯O1^i^	0.99	2.42	3.3096 (15)	149
C13—H13⋯O1^ii^	0.95	2.34	3.2252 (17)	156

Symmetry codes: (i) 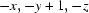; (ii) 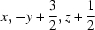.

**Fig. 1.**
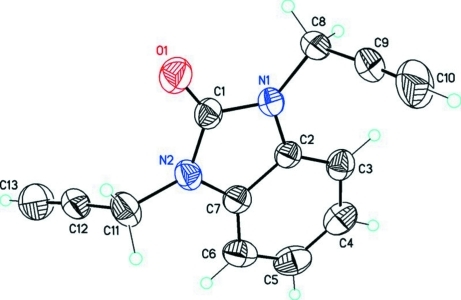
Molecular structure of the title compound with displacement ellipsoids drawn at the 50% probability level. H atoms are represented as small circles.

**Fig. 2.**
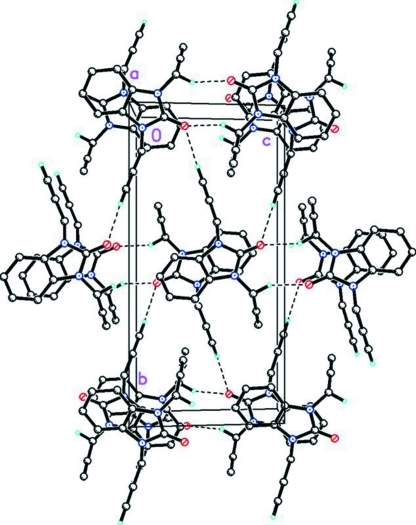
Partial packing view showing the C—H···O interactions (dashed lines).

## supplementary crystallographic information

### Comment

Benzimidazoles are very useful intermediates/subunits for the development of molecules of pharmaceutical or biological interest. Benzimidazole and its derivatives are an important class of bioactive molecules in the field of drugs and pharmaceuticals.

Benzimidazole derivatives have found applications in diverse therapeutic areas including anti-ulcers, anti-hypertensives, anti-virals, anti-fungals, anti-cancers (Gravatt *et al.*, 1994; Horton *et al.*, 2003; Kim *et al.*, 1996; Roth *et al.*, 1997).

As a continuation of our research works devoted to the development benzimidazol-2-one derivatives (Ouzidan *et al.*, 2011*a*,*b*), we report in this paper the synthesis of a new benzimidazol-2-one derivative prepared by action of propargyl bromide on 1*H*-benzimidazol-2(3*H*)-one in the presence of a catalytic quantity of tetra-n-butylammonium bromide under mild conditions to furnish the title compound (Scheme 1).

In the title compound (Fig. 1), the benzimidazole ring system is essentially planar with a maximum deviation of 0.015 (1) Å for C1 atom. The two propynyl chains are almost perpendicular to the benzimidazole mean plane but oriented one above and one below the plane. The molecular conformation is also characterized by the following torsion angles: C1-N1-C8-C9 = 93.5 (2) ° and C1-N2-C11-C12 = 105.9 (2) °. In the crystal structure, molecules are linked by weak intermolecular C—H···O no classic hydrogen bonds as shown in Fig. 2 and Table 2.

### Experimental

To a mixture of 1*H*-benzimidazol-2(3*H*)-one (0.2 g, 1.5 mmol), potassium carbonate (0.45 g, 3.2 mmol), tetra-n-butylammonium bromide (0.1 g, 0.2 mmol) in DMF (15 ml) was added propargyl bromide (0.28 ml, 3.2 mmol). Stirring was continued at room temperature for 6 h. The salt was removed by filtration and the filtrate concentrated under reduced pressure. The product was purified by recrystallization from dichloromethane to give colourless crystals (m.p. 425 K).

### Refinement

H atoms were located in a difference map and treated as riding with C—H = 0.95 Å or 0.99 Å  with *U*_iso_(H) = 1.2 *U*_eq_ (C).

### Crystal data

**Table:**

C_13_H_10_N_2_O	*F*(000) = 440
*M**_r_* = 210.23	*D*_x_ = 1.262 Mg m^−^^3^
Monoclinic, *P*2_1_/*c*	Mo *K*α radiation, λ = 0.71073 Å
Hall symbol: -P 2ybc	Cell parameters from 3141 reflections
*a* = 7.7398 (4) Å	θ = 3.4–32.2°
*b* = 17.1869 (9) Å	µ = 0.08 mm^−^^1^
*c* = 8.4856 (5) Å	*T* = 170 K
β = 101.459 (6)°	Block, colorless
*V* = 1106.28 (10) Å^3^	0.42 × 0.41 × 0.20 mm
*Z* = 4	

### Data collection

**Table:**

Oxford Diffraction Xcalibur E Gemini diffractometer	2631 independent reflections
Radiation source: Enhance (Mo) X-ray Source	2244 reflections with *I* > 2σ(*I*)
graphite	*R*_int_ = 0.014
Detector resolution: 16.1500 pixels mm^-1^	θ_max_ = 27.9°, θ_min_ = 3.4°
ω scans	*h* = −10→10
Absorption correction: multi-scan (*CrysAlis PRO*; Oxford Diffraction, 2009)	*k* = −20→22
*T*_min_ = 0.966, *T*_max_ = 0.984	*l* = −11→4
5295 measured reflections	

### Refinement

**Table:**

Refinement on *F*^2^	Secondary atom site location: difference Fourier map
Least-squares matrix: full	Hydrogen site location: inferred from neighbouring sites
*R*[*F*^2^ > 2σ(*F*^2^)] = 0.040	H-atom parameters constrained
*wR*(*F*^2^) = 0.104	*w* = 1/[σ^2^(*F*_o_^2^) + (0.0447*P*)^2^ + 0.2172*P*] where *P* = (*F*_o_^2^ + 2*F*_c_^2^)/3
*S* = 1.05	(Δ/σ)_max_ = 0.001
2631 reflections	Δρ_max_ = 0.19 e Å^−^^3^
146 parameters	Δρ_min_ = −0.16 e Å^−^^3^
0 restraints	Extinction correction: *SHELXL97* (Sheldrick, 2008), Fc^*^=kFc[1+0.001xFc^2^λ^3^/sin(2θ)]^-1/4^
Primary atom site location: structure-invariant direct methods	Extinction coefficient: 0.042 (4)

### Special details

**Table:**

Geometry. All e.s.d.'s (except the e.s.d. in the dihedral angle between two l.s. planes) are estimated using the full covariance matrix. The cell e.s.d.'s are taken into account individually in the estimation of e.s.d.'s in distances, angles and torsion angles; correlations between e.s.d.'s in cell parameters are only used when they are defined by crystal symmetry. An approximate (isotropic) treatment of cell e.s.d.'s is used for estimating e.s.d.'s involving l.s. planes.
Refinement. Refinement of *F*^2^ against ALL reflections. The weighted *R*-factor *wR* and goodness of fit *S* are based on *F*^2^, conventional *R*-factors *R* are based on *F*, with *F* set to zero for negative *F*^2^. The threshold expression of *F*^2^ > σ(*F*^2^) is used only for calculating *R*-factors(gt) *etc*. and is not relevant to the choice of reflections for refinement. *R*-factors based on *F*^2^ are statistically about twice as large as those based on *F*, and *R*- factors based on ALL data will be even larger.

### Fractional atomic coordinates and isotropic or equivalent isotropic displacement parameters (Å^2^)

**Table:**

	*x*	*y*	*z*	*U*_iso_*/*U*_eq_	
O1	0.19931 (12)	0.56190 (5)	0.15005 (11)	0.0468 (2)	
N1	0.13961 (12)	0.46462 (5)	0.32281 (11)	0.0338 (2)	
N2	0.29126 (12)	0.56670 (5)	0.42832 (12)	0.0356 (2)	
C1	0.20862 (14)	0.53446 (6)	0.28398 (14)	0.0346 (3)	
C2	0.18290 (13)	0.45232 (6)	0.48810 (13)	0.0319 (2)	
C3	0.14722 (15)	0.39128 (7)	0.58201 (15)	0.0400 (3)	
H3A	0.0826	0.3470	0.5362	0.048*	
C4	0.21003 (18)	0.39733 (9)	0.74692 (16)	0.0493 (3)	
H4A	0.1871	0.3565	0.8153	0.059*	
C5	0.30518 (18)	0.46160 (9)	0.81362 (16)	0.0524 (4)	
H5A	0.3463	0.4638	0.9267	0.063*	
C6	0.34172 (16)	0.52287 (8)	0.71876 (15)	0.0447 (3)	
H6A	0.4073	0.5669	0.7646	0.054*	
C7	0.27878 (14)	0.51721 (6)	0.55512 (14)	0.0337 (3)	
C8	0.06303 (16)	0.40760 (7)	0.20295 (15)	0.0398 (3)	
H8A	0.0159	0.4343	0.1000	0.048*	
H8B	−0.0362	0.3810	0.2383	0.048*	
C9	0.19408 (17)	0.34996 (7)	0.17828 (16)	0.0438 (3)	
C10	0.3035 (2)	0.30587 (10)	0.1615 (2)	0.0748 (5)	
H10	0.3927	0.2699	0.1479	0.090*	
C11	0.39412 (16)	0.63759 (7)	0.43751 (18)	0.0443 (3)	
H11A	0.4208	0.6484	0.3302	0.053*	
H11B	0.5076	0.6293	0.5131	0.053*	
C12	0.30770 (17)	0.70582 (7)	0.48968 (16)	0.0454 (3)	
C13	0.2472 (2)	0.76260 (9)	0.5312 (2)	0.0678 (5)	
H13	0.1980	0.8088	0.5649	0.081*	

### Atomic displacement parameters (Å^2^)

**Table:**

	*U*^11^	*U*^22^	*U*^33^	*U*^12^	*U*^13^	*U*^23^
O1	0.0561 (5)	0.0414 (5)	0.0426 (5)	0.0004 (4)	0.0090 (4)	0.0060 (4)
N1	0.0375 (5)	0.0275 (4)	0.0351 (5)	0.0017 (4)	0.0043 (4)	−0.0037 (4)
N2	0.0349 (5)	0.0290 (5)	0.0430 (5)	−0.0006 (4)	0.0080 (4)	−0.0051 (4)
C1	0.0339 (5)	0.0297 (5)	0.0405 (6)	0.0054 (4)	0.0079 (5)	−0.0013 (4)
C2	0.0276 (5)	0.0317 (5)	0.0363 (6)	0.0059 (4)	0.0060 (4)	−0.0026 (4)
C3	0.0359 (6)	0.0374 (6)	0.0481 (7)	0.0031 (5)	0.0118 (5)	0.0028 (5)
C4	0.0470 (7)	0.0577 (8)	0.0457 (7)	0.0070 (6)	0.0150 (6)	0.0114 (6)
C5	0.0489 (7)	0.0727 (9)	0.0351 (6)	0.0086 (7)	0.0068 (5)	0.0004 (6)
C6	0.0367 (6)	0.0538 (7)	0.0419 (7)	0.0025 (5)	0.0034 (5)	−0.0114 (6)
C7	0.0278 (5)	0.0348 (5)	0.0390 (6)	0.0055 (4)	0.0077 (4)	−0.0044 (4)
C8	0.0396 (6)	0.0349 (6)	0.0413 (6)	−0.0008 (5)	−0.0003 (5)	−0.0066 (5)
C9	0.0525 (7)	0.0328 (6)	0.0440 (7)	−0.0025 (5)	0.0043 (6)	−0.0082 (5)
C10	0.0733 (11)	0.0515 (9)	0.0976 (14)	0.0171 (8)	0.0124 (10)	−0.0250 (9)
C11	0.0358 (6)	0.0355 (6)	0.0628 (8)	−0.0057 (5)	0.0128 (6)	−0.0079 (6)
C12	0.0447 (7)	0.0340 (6)	0.0536 (8)	−0.0026 (5)	0.0002 (6)	−0.0054 (5)
C13	0.0771 (11)	0.0436 (8)	0.0749 (11)	0.0136 (7)	−0.0038 (9)	−0.0170 (7)

### Geometric parameters (Å, °)

**Table:**

O1—C1	1.2194 (14)	C5—H5A	0.9500
N1—C1	1.3800 (14)	C6—C7	1.3806 (17)
N1—C2	1.3919 (14)	C6—H6A	0.9500
N1—C8	1.4515 (14)	C8—C9	1.4622 (17)
N2—C1	1.3802 (15)	C8—H8A	0.9900
N2—C7	1.3899 (15)	C8—H8B	0.9900
N2—C11	1.4491 (14)	C9—C10	1.166 (2)
C2—C3	1.3782 (16)	C10—H10	0.9500
C2—C7	1.3965 (15)	C11—C12	1.4615 (17)
C3—C4	1.3911 (19)	C11—H11A	0.9900
C3—H3A	0.9500	C11—H11B	0.9900
C4—C5	1.385 (2)	C12—C13	1.1665 (19)
C4—H4A	0.9500	C13—H13	0.9500
C5—C6	1.388 (2)		
			
C1—N1—C2	110.22 (9)	C7—C6—H6A	121.4
C1—N1—C8	122.92 (10)	C5—C6—H6A	121.4
C2—N1—C8	125.91 (9)	C6—C7—N2	132.11 (11)
C1—N2—C7	110.49 (9)	C6—C7—C2	121.24 (11)
C1—N2—C11	122.56 (10)	N2—C7—C2	106.65 (10)
C7—N2—C11	126.50 (10)	N1—C8—C9	111.07 (10)
O1—C1—N1	127.23 (11)	N1—C8—H8A	109.4
O1—C1—N2	127.08 (11)	C9—C8—H8A	109.4
N1—C1—N2	105.69 (10)	N1—C8—H8B	109.4
C3—C2—N1	131.49 (11)	C9—C8—H8B	109.4
C3—C2—C7	121.59 (11)	H8A—C8—H8B	108.0
N1—C2—C7	106.92 (9)	C10—C9—C8	177.43 (15)
C2—C3—C4	117.04 (12)	C9—C10—H10	180.0
C2—C3—H3A	121.5	N2—C11—C12	114.29 (10)
C4—C3—H3A	121.5	N2—C11—H11A	108.7
C5—C4—C3	121.47 (12)	C12—C11—H11A	108.7
C5—C4—H4A	119.3	N2—C11—H11B	108.7
C3—C4—H4A	119.3	C12—C11—H11B	108.7
C4—C5—C6	121.41 (13)	H11A—C11—H11B	107.6
C4—C5—H5A	119.3	C13—C12—C11	176.21 (15)
C6—C5—H5A	119.3	C12—C13—H13	180.0
C7—C6—C5	117.25 (12)		
			
C2—N1—C1—O1	177.95 (11)	C4—C5—C6—C7	0.21 (19)
C8—N1—C1—O1	8.50 (18)	C5—C6—C7—N2	−179.89 (11)
C2—N1—C1—N2	−1.76 (11)	C5—C6—C7—C2	−0.23 (17)
C8—N1—C1—N2	−171.22 (9)	C1—N2—C7—C6	178.83 (11)
C7—N2—C1—O1	−178.10 (11)	C11—N2—C7—C6	6.44 (19)
C11—N2—C1—O1	−5.36 (18)	C1—N2—C7—C2	−0.87 (12)
C7—N2—C1—N1	1.61 (12)	C11—N2—C7—C2	−173.26 (10)
C11—N2—C1—N1	174.35 (9)	C3—C2—C7—C6	−0.08 (16)
C1—N1—C2—C3	−178.62 (11)	N1—C2—C7—C6	−179.97 (10)
C8—N1—C2—C3	−9.55 (18)	C3—C2—C7—N2	179.66 (10)
C1—N1—C2—C7	1.26 (11)	N1—C2—C7—N2	−0.23 (11)
C8—N1—C2—C7	170.33 (10)	C1—N1—C8—C9	93.46 (13)
N1—C2—C3—C4	−179.74 (11)	C2—N1—C8—C9	−74.31 (14)
C7—C2—C3—C4	0.40 (16)	C1—N2—C11—C12	105.88 (13)
C2—C3—C4—C5	−0.42 (18)	C7—N2—C11—C12	−82.58 (15)
C3—C4—C5—C6	0.1 (2)		

### Hydrogen-bond geometry (Å, °)

**Table:**

*D*—H···*A*	*D*—H	H···*A*	*D*···*A*	*D*—H···*A*
C8—H8A···O1^i^	0.99	2.42	3.3096 (15)	149
C13—H13···O1^ii^	0.95	2.34	3.2252 (17)	156

Symmetry codes: (i) −*x*, −*y*+1, −*z*; (ii) *x*, −*y*+3/2, *z*+1/2.

## References

[bb1] Gravatt, G. L., Baguley, B. C., Wilson, W. R. & Denny, W. A. (1994). *J. Med. Chem.* **37**, 4338–4345. 10.1021/jm00051a010 7527862

[bb2] Horton, D. A., Bourne, G. T. & Smythe, M. L. (2003). *Chem. Rev.* **103**, 893–930. 10.1021/cr020033s 12630855

[bb3] Kim, J. S., Gatto, B., Yu, C., Liu, A., Liu, L. F. & La Voie, E. J. (1996). *J. Med. Chem.* **39**, 992–998. 10.1021/jm950412w 8632422

[bb4] Ouzidan, Y., Kandri Rodi, Y., Butcher, R. J., Essassi, E. M. & El Ammari, L. (2011*a*). *Acta Cryst.* E**67**, o283. 10.1107/S1600536810054164 PMC3051596 21522975

[bb5] Ouzidan, Y., Kandri Rodi, Y., Fronczek, F. R., Venkatraman, R., El Ammari, L. & Essassi, E. M. (2011*b*). *Acta Cryst.* E**67**, o362–o363. 10.1107/S1600536810052141 PMC3051530 21523041

[bb6] Oxford Diffraction (2009). *CrysAlis PRO* Oxford Diffraction Ltd, Yarnton, England.

[bb7] Roth, T., Morningstar, M. L., Boyer, P. L., Hughes, S. H., Buckheit, R. W. & Michejda, C. J. (1997). *J. Med. Chem.* **40**, 4199–4207. 10.1021/jm970096g 9435891

[bb8] Sheldrick, G. M. (2008). *Acta Cryst.* A**64**, 112–122. 10.1107/S0108767307043930 18156677

